# Reference Data on Neonatal Serum N-Acetyl-*β*-hexosaminidase Activity

**DOI:** 10.1155/2018/6187245

**Published:** 2018-07-02

**Authors:** Sylwia Chojnowska, Monika Kamianowska, Piotr Baran, Krzysztof Zwierz, Marek Szczepański

**Affiliations:** ^1^Faculty of Health Sciences, Lomza State University of Applied Sciences, Łomża, Poland; ^2^Department of Neonatology and Newborn Intensive Care Unit, Medical University of Bialystok, Białystok, Poland; ^3^Clinic of Obstetrics and Gynecology, Bielański Hospital, Warsaw, Poland

## Abstract

**Background:**

Determination of neonate serum's N-acetyl-*β*-hexosaminidase (HEX) activity and correlation results with Apgar scale and factors routinely determined in newborn serum.

**Aims:**

Providing reference values of neonates serum HEX activities, and indicate their diagnostic significance.

**Study design:**

The study was performed using random serum samples of 111 infants (53 ♂/58 ♀), aged 1–30 days. The activity of HEX was determined colorimetrically and expressed in nKat/L.

**Results:**

Serum HEX activity of 111 newborns was 360.5 ± 114.0 nKat/L and significantly positively correlated with gestation week at the day of delivery, birth weight, weight on day of blood collection, sex, and serum CRP.

**Conclusions:**

Reference values presented for neonatal serum activities of HEX may be used in neonatal diagnostics, for example, to detect inflammation and other diseases or for early assessment of the risk of Tay-Sachs and Sandhoff diseases.

## 1. Introduction

Our paper is devoted to glycoconjugate catabolism [1]. Glycoconjugates comprise glycoproteins and proteoglycans with oligosaccharide and glycosaminoglycan chains, respectively, attached to polypeptides as well as glycolipids with oligosaccharide chains attached to lipids. Due to the hydrophilicity of oligosaccharide and glycosaminoglycan chains, glycan moieties regulate the glycoproteins and proteoglycans folding steady-state cellular distribution, stability, and functions [2, 3]. Glycoconjugates are main components of extracellular matrix and glycocalyx of the cell surface, where they mediate cell-cell recognition and interaction with pathogens, hormones, and toxins [4–7]. Glycoconjugates undergo permanent renovation. Old glycoconjugate molecules are degraded, and on their place are synthesized new ones. Catabolism of glycoconjugates occurs mainly in lysosomes, where about 50 hydrolytic enzymes and among them exoglycosidase are located. Exoglycosidases hydrolyse O-glycosidic linkages releasing monosugars attached to the sides or nonreducing ends of oligosaccharide chains [8]. Lysosomal exoglycosidases and among them N-acetyl-*β*-hexosaminidase (HEX), the most active of lysosomal exoglycosidases, reflect the intensity of tissue breakdown and remodeling [9, 10]. Deficiencies of N-acetyl-*β*-hexosaminidase A and B isoenzyme activities in neonatal body are the reason for storing gangliosides in their lysosomes that cause Sandhoff and Tay-Sachs diseases [11]. HEX high activity in serum reflects high intensity of tissue glycoconjugate degradation caused by the inflammatory conditions and other etiologies [12]. Increased HEX activity has been found in the urine of neonates with nephrotoxic drug intoxication [13], fever [14], and in the urine of children with nephritis [15] as well as solitary functioning kidney [16]. Lobe et al. [17] suggested determination of the serum HEX activity for early diagnosis of the necrotizing enterocolitis (NEC) in the premature babies, but Shattuck et al. [18] claim that serum HEX activity concentration is not a good marker for NEC because reference ranges in newborn children have not been properly characterized. Therefore, for more precise diagnostics of disturbances in newborn glycoconjugate catabolism, we decided to determine reference values of HEX in the neonate serum and correlate them with other parameters determined routinely in neonate serum.

## 2. Materials and Methods

### 2.1. Ethics

Approval for this study was granted by the Ethics Review Board of the Medical University of Bialystok (R-I-002/440/2014.).

### 2.2. Blood Collection

The study material consisted of 111 serum samples which were taken from neonates for routine laboratory testing. In the study group, there were 58 girls and 53 boys, aged 1–30 days (mean 6.4 ± 7.6 days) born between 26 and 41 weeks of gestation (mean 35.5 ± 3.6), with birth weight of 650–4560 g (mean 2471.8 ± 973 g). The body weight on the day of blood collection varied between 710 and 4100 g (mean 2491 ± 886.7 g). In the hospital, laboratory serum routine biochemical parameters were determined including bilirubin and CRP. After performance routine biochemical tests, the remaining neonatal serum samples were stored at −80°C for the determination of HEX activity.

### 2.3. Determination of HEX Activity in Neonate Serums

Taking into account the increased level of bilirubin in neonatal serum, HEX determination was performed by the recently modified method of Chojnowska et al. [19] as follows: to 10 *μ*L of neonatal serum with bilirubin was added 40 *μ*L 0.1 mol/L citrate-phosphate buffer pH 4.7 and 30 *μ*L 6.7 mmol/L 4-nitrophenyl-N-acetyl-*β*-glucosaminide (Sigma-Aldrich, St. Louis, USA) as a substrate. The reaction mixture was incubated at 37°C for 60 min (Thermo Shaker Incubator DTS4, ELMI Ltd., Latvia). The enzymatic reaction was terminated by adding 200 *μ*L 0.2 mol/L borate buffer at pH 9.8. The liberated 4-nitrophenol was measured spectrophotometrically (Infinite ® 200 PRO, TECAN, Switzerland) at 410 nm using two blanks: the first blank contained substrate without serum (10 *μ*L distilled water + 40 *μ*L 0.1 mol/L citrate-phosphate buffer pH 4.7 + 30 *μ*L substrate + 200 *μ*L 0.2 mol/L borate buffer at pH 9.8); the second blank contained neonatal serum without substrate (10 *μ*L neonatal serum + 70 *μ*L 0.1 mol/L citrate-phosphate buffer pH 4.7 + 200 *μ*L 0.2 mol/L borate buffer pH 9.8). The HEX activity was read from the calibration curve (standard: 0.25 mmol/L 4-nitrophenol in 0.1 mol/L phosphate-citrate buffer, pH 4.7) and expressed as nKat/L of neonatal serum.

### 2.4. Statistical Analysis

The collected data was analyzed statistically using Statistica version 10.0 (StatSoft, Cracow, Poland) with the Spearman's correlation. A value of *p* < 0.05 was taken as being significant.

## 3. Results

**Figure 1:**
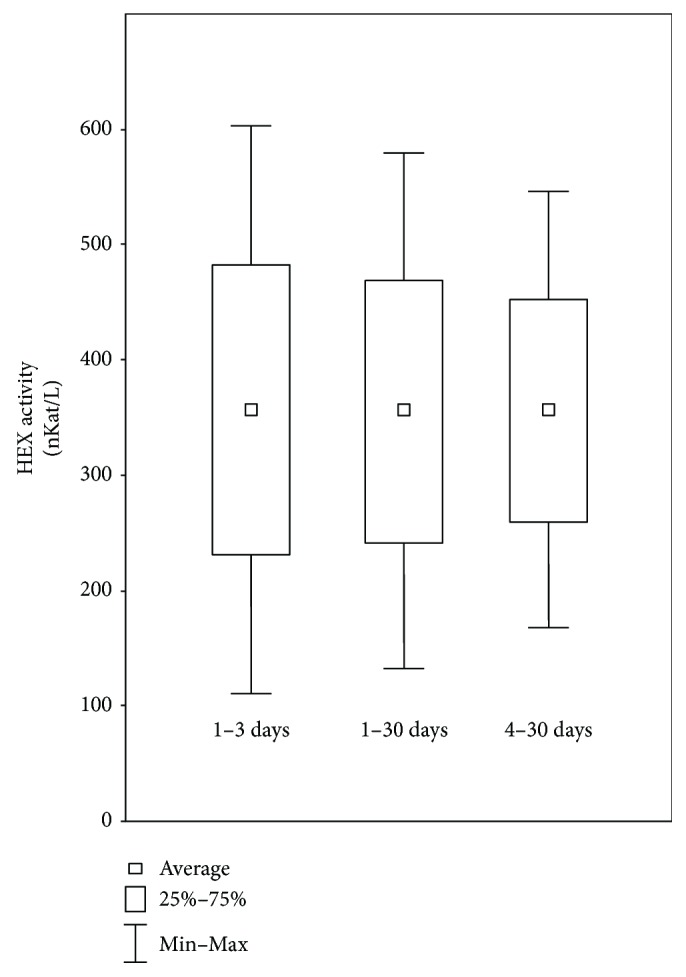
Changes of HEX activities in neonate serum during the first 30 days of life: the “1–3 days” bar presents activities of HEX in serum collected 1–3 days from delivery; the “1–30 days” bar presents activities of HEX in neonate serums collected 1–30 days from delivery; the “4–30 days” bar presents activities of HEX in serum collected 4–30 days from delivery.

**Figure 2:**
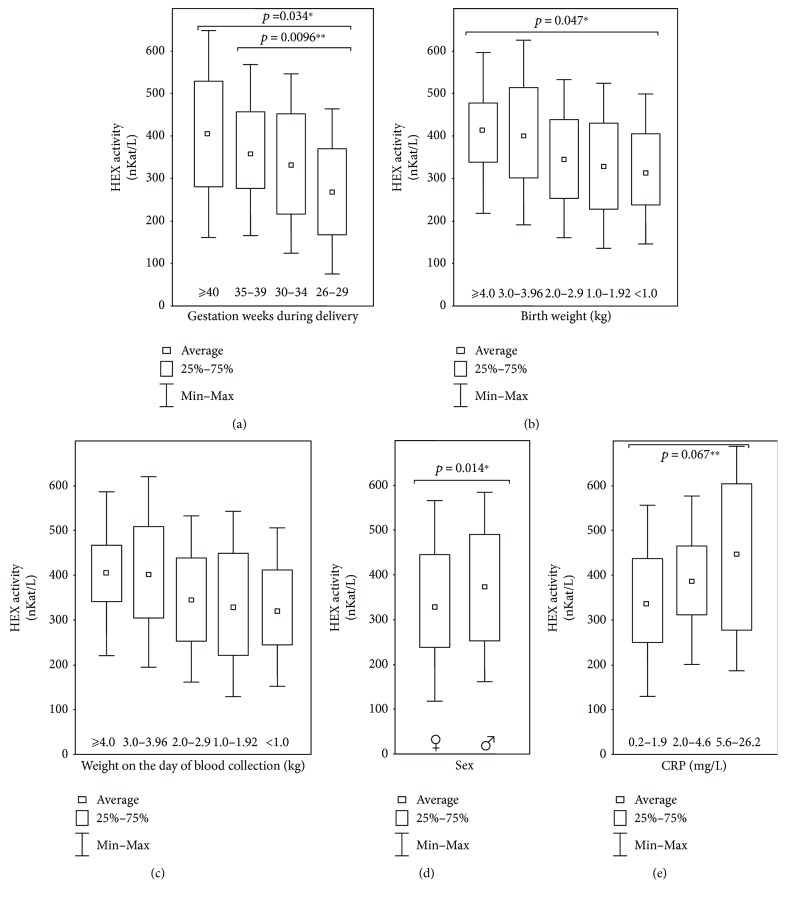
Relation between the neonate serum HEX activities and (a) gestation week during delivery, (b) birth weight, (c) weight during blood collection, (d) sex, and (e) neonate serum CRP concentration. (a) ≥40; 35–39; 30–34; and 26–29 bars specify neonate serum HEX activity in relation to maturity of neonates expressed in number of gestation weeks during delivery. (b) ≥4; 3.0–3.96; 2.0–2.9; 1.0–1.92; <1.0 bars specify neonate serum HEX activity in relation to neonate weight during delivery. (c) ≥4; 3.0–3. 96; 2.0–2.9; 1.0–1.92; <1.0 bars specify the relation of neonate serum HEX activity to neonate weight on the day of blood collection (kg); (d) ♀ and ♂ bars specify the relation of HEX activity in neonates serum during blood collection to sex. (e) 0.2–1.9; 2.0–4.6; and 5.6–26.2 bars specify the relation of neonate serum HEX activity to neonate serum CRP concentration in the same serums. Statistical significance: ^∗^*p* < 0.05, ^∗∗^*p* < 0.01.

In serums obtained from 111 neonates, HEX activity amounted 360.5 ± 114.0 nKat/L. Average HEX activity in the 1st day of neonatal life was similar to activity on the 30th day; however, on the 30th, day we observed lower standard deviations (Figure 1). The neonates' serum HEX activity significantly positively correlated with gestation weeks during delivery (*r* = 0.27; *p* = 0.0059^∗∗^), postnatal weight (*r* = 0.30; *p* = 0.0018^∗∗^), weight on the day of blood collection (*r* = 0.32; *p* = 0.0010^∗∗^), sex (*r* = 0.025; *p* = 0.0104^∗^), and CRP level in blood serum (*r* = 0.42; *p* = 0.00003^∗∗∗^) (Table 1). We did not find any significant correlation between neonates' serum HEX activity and Apgar's score determined on the day of delivery, day of life, and serum bilirubin concentration (Table 1). We checked also the relation among the neonate serum HEX activities and gestation week during delivery (Figure 2(a)), birth weight (Figure 2(b)), weight during blood collection (Figure 2(c)), sex (Figure 2(d)) and serum's CRP concentration (Figure 2(e)). We found statistically significant decrease of the serum HEX activity of the neonates born at 26–29 weeks (*p* = 0.034^∗^) and 35–39 weeks (*p* = 0.0096^∗∗^) of pregnancy, in comparison to neonates delivered at ≥40 weeks (Figure 2(a)), with the lightest body weight (<1.0 kg body weight) in comparison to the heaviest neonates (≥4.0 kg body weight) (*p* = 0.047^∗^) (Figure 2(b), 2(c)), girls in comparison to boys (*p* = 0.014^∗^) (Figure 2(d)), and at 0.2–2.9 mg/L in comparison to 5.6–26.2 mg/L serum's CRP levels (*p* = 0.0067^∗∗^) (Figure 2(e)).

## 4. Discussion

Exoglycosidase activities engaged in glycoconjugate catabolism are utilized in diagnostics of adults [10, 12], children [15, 16], and neonates [13, 14, 17, 18]. Deficiencies of N-acetyl-*β*-hexosaminidase A and B isoenzyme activities in neonatal body are the reason for storing glycoconjugates in their lysosomes that cause Sandhoff and Tay-Sachs diseases [11]. The standard procedure for the final detection of Tay-Sachs and Sandhoff diseases involves a measurement of the HEX activity in tissue cells, leucocytes, and serum [20–22]. In our experience [23], searching for Tay-Sachs disease should be started from blood serum because the determination of HEX in serum, which depend on HEX activity in tissues, is more convenient than the HEX determination in cells or cell organelles. In the Tay-Sachs disease, a lack of isoenzyme A (*αβ*) in tissues and serum activity is observed, which is caused by mutation in α subunit. However, activity of HEX B (*ββ*) still remains [24]. When the serum HEX activity is close to null, it is reasonable to measure the HEX activity in the materials that have the highest possible specific activity of HEX, for example, leucocytes and tissue cells. According to Zwierz et al. [8], the best material to search for HEX activity is the cell lysosome, as specific HEX activity in lysosomes, for example, human gastric mucous membrane, is 8 times higher than in cytoplasm and 12 times higher than in microsomes. For differentiation of HEX isoenzyme A from isoenzyme B, Borzym-Kluczyk et al. recommend electrophoretic separation of HEX isoenzymes [25]. The determination of HEX in neonate serum and leucocytes or fibrocytes with Tay-Sachs disease (lack of isoenzyme A of the HEX) will be very difficult in Poland and Europe because the frequency of Tay-Sachs disease in the world is 1 : 200,000 [26]. Only French Canadians living in eastern Quebec suffer from Tay-Sachs disease 10 times more often than the general population [27]. Additionally, among Ashkenazi Jews living mostly in the USA, the frequency of Tay-Sachs disease amounted to 1 : 3900 [26], and the frequency of Tay-Sachs carriers amounted to 1 : 25 [24].

Concerning HEX as a biomarker of neonate diseases, we are taking into consideration neonatal clinical situations related to an increase in CRP—perinatal asphyxia, meconium aspiration pneumonitis, intraventricular hemorrhage, fetal distress, and shock; serum AST and ALT activity—congenital infections (e.g., cytomegalovirus, rubella virus, Toxoplasma gondi, herpes simplex), hypoxic hepatic injury, cholestasis (e.g., biliary atresia, neonatal hepatitis, Alagille syndrome, and parenteral nutrition), and inherited metabolic diseases (e.g., galactosemia); and free radicals—retinopathy of prematurity (ROP), periventricular leukomalacia (PVL), bronchopulmonary dysplasia (BPD), respiratory distress syndrome (RDS), necrotizing enterocolitis (NEC), and intraventricular hemorrhage (IVH), which are the most frequent in the neonatal period. However, frequency of using exoglycosidase activity of neonatal tissues and body fluids for neonatal diagnostics is insufficient because of the lack of suitable reference data. Therefore, the aim of our present work is to provide data on the activity of HEX in neonatal serums.

We have stated that during the duration of pregnancy, activity of HEX in neonate serum (Figure 2(a)) increased significantly (*p* = 0.0096^∗^). In preterm infants (26–29 weeks of pregnancy), serum HEX activity amounted to 267 nKat/L, but in newborns about 40 weeks of gestation, serum's HEX activity amounted to 415 nKat/L (Figure 2(a)). Increase of HEX activity in neonate serum correlated positively with birth weight and weight during blood collection for HEX determination (Table 1, Figure 2(b)). In our study, an increase in neonates' weight positively correlates with intensity of glycoconjugate catabolism as we have found significant increase in HEX activity in heavier neonates (Figure 2(b)). Increase in HEX activity may also be related to newborn thymus weight and the secretion of HEX from the thymus (an especially active organ in neonates) to serum. According to Platt and Hartmann [28], specific activity of HEX in the human thymus is 3 times higher than in the human liver, which has to be considered when discussing the origin of HEX in neonate serum.

The data about significant higher serum HEX activity (388.2 ± 113.0 nKat/L) of male neonates in comparison to female neonates (335.3 ± 110.0 nKat/L) (*p* = 0.014^∗^) (Figure 2(d)) are in agreement with reports of other authors [29–32]. Differences in neonate serum HEX activity between males and females may depend on weight, but influence of sex hormones (mainly testosterone) may also be taken into consideration [31]. We also found a positive significant (*p* = 0.00003^∗∗∗^) correlation between neonatal serum HEX activity and concentration of serum CRP (Table 1, Figure 2(e)) that may be explained by the increase of serum HEX and CRP activities in infections and inflammations [10, 12, 14].

Our results are compatible with the report of Agirbasli et al. [32], who proved that urinary HEX excretion by young people (18–32 years old) was dependent on age, gender, race, and blood pressure. Recently Zalewska-Szajda et al. [33] also reported that reference activities of urinary HEX in children and adolescents are significantly dependent on age but not gender.

In the near future, we plan to study the activity of HEX and other exoglycosidases in the serum and urine of neonates suffering from disorders that are expected to increase activities in HEX and other exoglycosidases.

## 5. Conclusions

Reported data present reference values for activities of HEX in neonatal serums. Deviations from the reference range of neonatal serum HEX activity may be used for an early assessment of inherited storage disorders risks (Tay Sachs/Sandhoff diseases). The determination of HEX activities in neonatal serum may be helpful in the diagnosis of diseases accompanied by increasing serum CRP concentration, AST and ALT activities, and/or tissues free radicals.

## Figures and Tables

**Table 1:** Spearman's correlations between activity of the neonate serum HEX with other parameters.

HEX activity	Spearman's correlation coefficient	Statistical significance
Week of pregnancy in which neonate was born	*r* = 0.27	*p* = 0.0059^∗∗^
Day of life	*r* = 0.07	*p* = 0.4513
Apgar scale	*r* = 0.10	*p* = 0.2953
Birth weight	*r* = 0.30	*p* = 0.0018^∗∗^
Weight on day of blood collection	*r* = 0.32	*p* = 0.0010^∗∗^
Sex	*r* = 0.25	*p* = 0.0104^∗^
Serum bilirubin level	*r* = −0.04	*p* = 0.7222
Serum CRP level	*r* = 0.42	*p* = 0.00003^∗∗∗^

Statistical significance: ^∗^*p* < 0.05, ^∗∗^*p* < 0.01, ^∗∗∗^*p* < 0.001.

## Data Availability

No data was used to support this study.
